# A Modified Decision Tree Algorithm Based on Genetic Algorithm for Mobile User Classification Problem

**DOI:** 10.1155/2014/468324

**Published:** 2014-02-09

**Authors:** Dong-sheng Liu, Shu-jiang Fan

**Affiliations:** ^1^College of Computer Science & Information Engineering, Zhejiang Gongshang University, Hangzhou 310018, China; ^2^Center for Studies of Modern Business, Zhejiang Gongshang University, Hangzhou 310018, China

## Abstract

In order to offer mobile customers better service, we should classify the mobile user firstly. Aimed at the limitations of previous classification methods, this paper puts forward a modified decision tree algorithm for mobile user classification, which introduced genetic algorithm to optimize the results of the decision tree algorithm. We also take the context information as a classification attributes for the mobile user and we classify the context into public context and private context classes. Then we analyze the processes and operators of the algorithm. At last, we make an experiment on the mobile user with the algorithm, we can classify the mobile user into Basic service user, E-service user, Plus service user, and Total service user classes and we can also get some rules about the mobile user. Compared to C4.5 decision tree algorithm and SVM algorithm, the algorithm we proposed in this paper has higher accuracy and more simplicity.

## 1. Introduction

With the rapid development of mobile internet, mobile users can enjoy mobile services at anytime from anywhere, such as location-based services, mobile games, location-based advertising, and mobile phone rescue. By the end of March 2013, the number of mobile communication service users in China has reached 1.146 billion, which is 1.24% higher than last month and 12.46% higher than the same period last year. Facing the huge number of users, how to provide personalized services to customers, and how to make customer classification to mobile users based on data mining technologies have become the focus of the current academic and industry attention.

There are many methods which have been used to classify the customer. Han et al. [1] segmented the telecom customers based on customer value by decision tree model, they proposed a novel customer segmentation method based on customer lifecycle, and a decision tree method was developed to extract important parameters of customer value. In this study, the authors only took the customer value into consideration and did not take the social attribute of the user into consideration. Xiao et al. [2] proposed a dynamic classifier ensemble method for imbalanced data. Bayesian network was also used as a tool to the customer classification [3].

In generally, the Bayes classifier is not as sensitive as the C4.5 (one of decision tree algorithm) classifier [4]; compared to neural network, the decision tree has a better quality to deal with the nonnumeric data and can be understand easier; neural network needs many parameters when running it and has a long time to learning [5]; support vector machine classifier has a high precision but the result cannot be understood easily. So we select decision tree as a tool to generate rules in this paper. But most of the decision tree algorithms are greedy algorithm; greedy algorithm is usually running fast, but it does not get the optimal decision tree. To get optimal decision tree problem is NP complete problem; these methods cannot solve it. This paper puts forward a new decision model for mobile user classification, which introduced genetic algorithm to optimize the results of the decision tree algorithm.

## 2. Related Work

### 2.1. Classification Model

There are many classification models which have been proposed by researchers, such as decision tree algorithm, Bayesian network, genetic algorithm, and neural net algorithm.

Zhang [6] took the annual salary, education, age, occupation, marriage, and property attributes of customer as the decision attribute set and established the classification model for Chinese customers of bank based on decision tree. She has classified the customer into risk customer, bad customer, ordinary customer, and important customer classes. She used a single data mining method and used it in bank customer classification. The accuracy of the result may not be very accurate, so it will not be suitable to the mobile user classification.

Chen [7] proposed a tree classification model based on Bayesian network algorithm. This model which the researcher proposed uses a single method to classify the trees, which may be very useful in small data sets. For big data sets, the accuracy of the model will decrease. Moreover, as we mentioned before, the Bayesian classifier is not as sensitive as decision tree classifier.

Zhou et al. proposed a data selection model based on neural network [8]; this model used a modified neural network to constructed the classifier; it may be very useful in reducing time consume, but the accuracy may be not very satisfactory, and the model also used a single method to problem. Moreover as we mentioned before, neural network has a bad quality to deal with the nonnumeric data and low learning rate.

Shu [9] proposed a fingerprint classification system based on a modified genetic algorithm. In this study, an improvement of the born classification is designed by adding a joined BP operator GA; it may suit the fingerprint classification, but it is not very useful to mobile user data.

Zhou has put the applied the SVM in mobile communication churn and got a better result. But as we mentioned before, support vector machine classifier has a high precision but the result cannot be understood easily.

Most of these studies are based on a single data mining technique. There have been few attempts to apply several techniques simultaneously and combine their outcomes for classification model and it is not very useful to mobile user classification.

### 2.2. Decision Tree Algorithm

The classical decision tree algorithm includes ID3 algorithm [10], C4.5 algorithm based on ID3 algorithm [11], CHAID algorithm (CHI-squared Automatic Interaction Detector) [12], and CART algorithm (Classification and Regression Tree) [13].

C4.5 algorithm is a modified algorithm based on ID3. Compared to ID3 algorithm, C4.5 algorithm can describe the continual attribute situation, but ID3 algorithm cannot. And C4.5 algorithm has a faster speed in realizing the process than ID3 algorithm. Moreover the decision tree structure of C4.5 is also more reasonable than ID3 algorithm and also finds the good rules information. Compared to CART decision tree algorithm, C4.5 can construct multitree and CART algorithm only construct binary tree.

As we all know C4.5 is a modified algorithm to generate decision tree based on ID3 algorithm. C5.0/See 5.0 is commercialized versions of C4.5; the core of C5.0/See 5.0 is the same with C4.5, but C5.0/See 5.0 has been modified in execution efficiency and memory. Based on C4.5, C5.0 algorithm not only includes all functions of C4.5, but also introduces many new technologies. Particularly, one of the most important technologies is boosting technique [14, 15] which further improve the recognition rate of the sample. Compared with C4.5, C5.0 with higher accuracy, faster running speed, and smaller decision tree model takes up less computer memory. Additionally, the character of C5.0 algorithm is low complexity, easily and high adaptability. Owning to the advantages of C5.0 algorithm, many scholars have applied the algorithm to a series of applications. For example, based on C5.0 algorithm, Pang and Gong researched personal credit evaluation on bank [16]. Taiwan scholar Chiang [4] classifies clients with C5.0 algorithm.

## 3. User Classification and Mobile User Classification

Normal classification model may not be suitable for the mobile user classification because of dynamic of the mobile user data and because the data is so large. We will analyze this in the follow sections.

### 3.1. User Classification

User classification or customer classification can be defined as verifying the identification and differentiation of customers based on customer attributes. The customer attributes usually include social attributes, behavior, and value attributes. Customer classification can analyze customer's consumption behavior and also can analyze the customer's consumption psychology. Companies can provide different products for different behavior patterns, for different consumer psychology of customers with different promotion methods, and so forth. Customer classification is the basis of the customer analysis, mining the data which are after the classification is more targeted and can get more meaningful results.

In generally, classification methods and cluster methods can be used to user classification. Classification methods, such as decision tree algorithm, neural network, and SVM method. Cluster methods can be described as clustering the user data, analyzing each cluster of the user, and summing up the similarity or some attributes in common in each cluster users.

Mobile user classification may differ with the general users, for it has more attributes, such as context attribute, huge number of user, and we will analysis it in the following section. This requires that the mobile user classification method has higher accuracy, andnormal method we mentioned before will not be suitable for the mobile user classification. So in this paper we proposed a modified decision tree algorithm for the mobile user classification.

### 3.2. Mobile User Classification

There are many works on mobile users, such as Yang and Fei who have researched on Broadcasting in vehicular networks [17] and many classification models for user. But these classification models always have their limitations, such as low accuracy and complex and low running speed.

In the mobile user data set, it always includes a lot of user's attributes, such as basic information about the user: age, income, hometown, education, and so on; other information such as consume information like basic cost and Wireless cost will also contained in the data set; mobile user information also including context information about the mobile user, such as the mobile user can request mobile service with “anytime, any where”, it is a dynamic data stream. So if we want to classify the mobile user precisely, we should take the context information into account. In this paper, we can use the context information as a classification attribute. We classify the context attribute into private context and public context classes. In this paper, we can define the private context as the environment information about a mobile user's stays in a private place himself, such as a small room, quiet and whether the user enjoys solitude. The public context can be defined as the environment information about a mobile user's stays in a crowd place; for example, a user stays in a bus station; he may request different services in a private room. In other words, under the different contexts, the user will have different requirements, and it will influence the classification result.

Another important thing is that not every attribute has equally weight to classify the mobile user. Although decision tree could select the main important attributes, the result may not be optimized. In addition, mobile users are a large number in any countries or cities. It can use the “Big data” to express it. So to classify the mobile user with high accuracy, it seems as a hard work to do.

As we analyze above, if we want to classify the mobile user with these numbers of attributes and huge number of users with high accuracy, these models we mentioned before seem not very suitable. So we will propose a new model for the mobile user classification which will classify mobile user into classes with high accuracy.

### 3.3. A Modified Mobile User Classification Model

**Figure 1:**
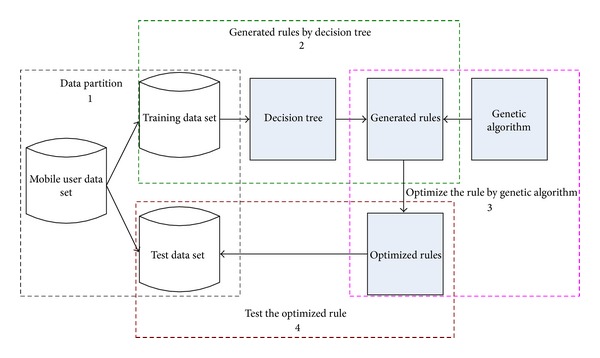
Framework of the proposed model.

As mentioned in the previous section, we propose a new customer classification mode based on decision tree and genetic algorithm. The overall framework of the proposed model is shown in Figure 1.

As is shown in Figure 1, the process of this model consists of four steps in total. The detail explanation for each step of the proposed model is presented as follows.

**Step 1**

*Data Partition*. In this process, we should partition the customer data; we partition the data into training data set and test data set. We can partition the data in percentage terms. For example, if there are *n* sets of data, we can take 70% × *n* of them as the training data and the else of data as the test data; this step will reduce the data amount and will also provide the test data set in the following step.

**Step 2**

*Generated Rules by Decision Tree*. In this step, we will use decision tree algorithm to the training data set to generate inference rules. The decision tree algorithm can be ID3 algorithm, CHAID algorithm (CHI-squared Automatic Interaction Detector), or C4.5 algorithm. In this paper, we take C4.5 algorithm as a tool to generate rules because of the accuracy and low complexity of the algorithm.

**Step 3**

*Optimize the Rule by Genetic Algorithm*. After generating rules, we should optimize the rule, because the rule may not bethe optimization for the data. In this paper, we use genetic algorithm to optimize the rule; we will analyze this step in the following section in detail.

**Step 4**

*Test the Optimized Rule*. In this step, we use the test data set to verify the accuracy of the optimized rule.

Through these steps, we can finally get the optimization rule for the data set. Steps 1, 2, and 4 are normal steps, so we will not describe them again; Step 3 is the main point of our paper, so we will describe it in detail in Section 4.

## 4. Modified Decision Tree for Mobile User Classification Based on Genetic Algorithm

### 4.1. The Basic Decision Tree Algorithm

**Figure 2:**
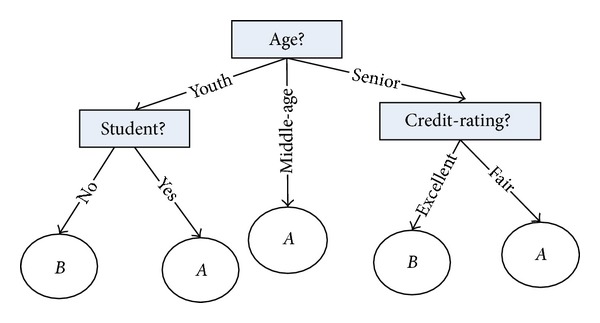
A sample decision tree.

A decision tree is a flow-chart-like tree structure, where each internal node (nonleaf node) denotes a test on attribute, each branch represents an outcome of the test, and each leaf node (or terminal node) holds a class label. The topmost node in a tree is the root node. A typical decision tree is shown in Figure 2. The decision tree algorithm usually has three popular attribute selection measures, namely, information gain, gain ratio, and gini index.

Assuming that *S* is the set of data samples, the attributes of class label have *m* different value, and the number of different classes *C*_*i*_  (*i* = 1,2,…, *m*) to be  *m*. Set *s*_*i*_ is the number of samples in class *C*_*i*_. For a given sample, the expected information needed for classification is given by the following equation:

(1) $$\begin{array}{c} I\left(s_{1},s_{2},\ldots ,s_{m}\right)=-\overset{m}{\underset{i=1}{\sum }}p_{i}\log_{2}\left(p_{i}\right), \end{array}$$

where *p*_*i*_ = *s*_*i*_/*s* is the probability of any sample belonging to *C*_*i*_.

Set attribute *A* with *v* different values {*a*_1_, *a*_2_,…, *a*_*v*_}. Then *S* could be divided into *v* subsets {*s*_1_, *s*_2,_ …, *s*_*v*_} by attribute *A*. Where the sample of *s*_*j*_ has the same value *a*_*j*_  (*j* = 1,2,…, *v*) in the attribute *A*. Set *s*_*ij*_ to be the number of the sample of class *C*_*i*_ in a subset *s*_*j*_. The entropy and information expectations of the subsets divided by *A* are given by the following expression

(2) $$\begin{array}{c} E\left(A\right)=\overset{v}{\underset{i=1}{\sum }}\frac{\left(s_{1j}+s_{2j}+\cdots +s_{mj}\right)}{s}I\left(s_{1j},s_{2j},\ldots ,s_{mj}\right). \end{array}$$

When the entropy value is smaller, the purity of subset partition will be higher. For a given subset *s*_*j*_, the expected information is:

(3) $$\begin{array}{c} I\left(s_{1j},s_{2j},\ldots ,s_{mj}\right)=-\overset{m}{\underset{i=1}{\sum }}p_{ij}\log_{2}\left(p_{ij}\right), \end{array}$$

where *p*_*ij*_ = *s*_*ij*_/*s*_*j*_ is the probability of the sample of *s*_*j*_ belonging to *C*_*i*_. If we conduct the branch operation in the attribute *A*, the information gain received is Gain(*A*) = *I*(*s*_1*j*_, *s*_2*j*_,…, *s*_*mj*_) − *E*(*A*). Then according to the split information Split_info(*A*) that is used to measure the breadth and uniformity of the split of data, the size of the information gain rate is compared in the process of the attribute classification; then the attribute with the maximum information gain rate is chosen for split attributes.

Where the split information and information gain ratio can be, respectively, expressed as

(4) $$\begin{array}{c} \text{Split}\_\text{info}\left(A\right)=\overset{v}{\underset{j=1}{\sum }}\left|\frac{s_{j}}{s}\right|\log\left(\left|\frac{s_{j}}{s}\right|\right),\\ \text{gain}\_\text{ration}\left(A\right)=\frac{\text{Gain}\left(A\right)}{\text{Split}\_\text{info}\left(A\right)}. \end{array}$$

Repeat the above steps until all the attributes are classified.

Decision tree cannot only construct the tree but also produce the inference rules. The description is shown as follows.

(5) IF  condition  1  and  condition  2  and  condition  3…and  condition  n  then  Class  A,

where *conditioni* is the preconditions and *A* is the class type. So we can see that classification rules are logic formulas who come from conjunctive normal form; the left of each rule conjunction item corresponds to the feature attributes. In Figure 2, we can get the following expressions as shown in Algorithm 1.

### 4.2. Modified Decision Tree Algorithm Based on Genetic Algorithm

As we mentioned before, decision tree always cannot get the optimize rule and genetic algorithm is usually used as a optimize tool. So we can use genetic algorithm to optimize the result of decision tree.

**Figure 3:**
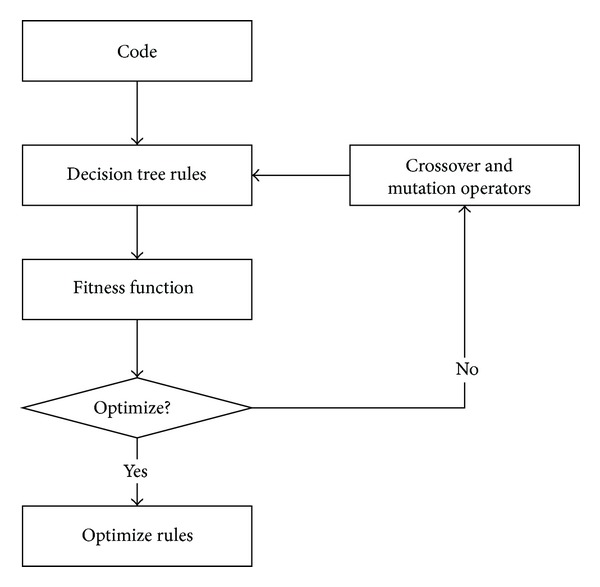
Processes of algorithm.

The idea of the algorithm we proposed in this paper is that we firstly use the decision tree algorithm to generate the mobile user classification rules, and then according to the attribute of the rule, such as accuracy, support, simplicity, and gain ratio, we construct the fitness function of genetic algorithm. The larger the value of the fitness is, the more the optimal rule will be. We use the crossover operation and mutation operation of genetic to adjust the fitness function, so the fitness value will reach to the maximum value, and the rule will be optimization. The processes are shown in Figure 3. We will describe these steps in following sections.

#### 4.2.1. Coding for the Rule

In general, genetic algorithm adopts bit coding with fixed length; the most common of use is the binary code; this method use a string which is constituted by the symbol {0, 1} to denote an individual. Each code responding to a condition attribute and the attribute value will determine the encoding length. For example, an attribute has *K* kinds of value (the continuous attributes need discretized firstly), so the individual coding will distribute *K* bits for it and each bit corresponding the possible values. When the value is 0, it means that the individual will not take the attribute value. When the value is 1, the individual will take the attribute value. Transformation of this method is simple and each chromosome has fixed length. However, the decision tree has a feature that the node has not only discrete attributes, but also numerical attributes. The simple binary code is not very useful.

In this paper, we set that each chromosome represents a classification rule. Some chromosomes will become the solution of problem. The final rule set will be sorted by the quality of the rule. When the rule set is used to recognize a new sample, the best rule will be considered firstly; if the best rule cannot recognize the sample, then we can choose the next rule. If the rule in the rule set cannot recognize the sample either, the sample will be classified as default class. Chromosomes will compete with each other in priority of the population.

Assume that the data include *n* attributes, so each chromosome will be divided into *n* genes, the *i*th gene corresponding to the *i*th attribute. Each individual represents a classification rule and each gene represents the left side of classification rule or the right side. The whole chromosome can represent a completed rule *IF-THEN*. The left side of classification rule is constructed by the genes which correspond to the characteristic attributes; we called these genes as characteristic genes; the right side of the rule is constructed by genes which correspond to class attribute; we called this gene will as class gene. During the gene evolution, the characteristic genes will participate in the evolution, but the class gene not. Each chromosome has a fixed length and has some genes. Inner of each gene includes four parts: {Weight, Operator, Value, Gain  ratio}.

*Weight.* Weight is a Boolean variable; it represents weather gene which corresponds to the attribute appears; if the weight is 1, the attribute which corresponds to the gene will appear in the rule. On the contrary, the weight is 0, which means that the attribute which corresponds to the gene will not appear in the rule.

*Operator.* It denotes the operators that genes conjunction adopt. To the discrete attributes, the value should be “=” or “≠”; the continuous attributes, the value, should be “≥” or “≺”.

*Value.* Value denotes the value of the attribute. To the discrete attributes, the value equals the site where actual values in the domain of value. To the continuous attributes value is equal to the actual value.

**Figure 4:**
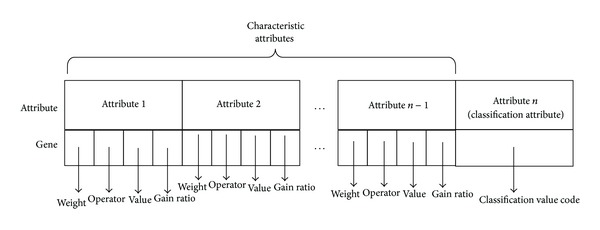
Chromosome construction.

*Gain Ratio.* It denotes the information gain rate of the attribute; it can be calculated with the formula in Section 2. Before the genetic algorithm begins, calculate and save all attributes information gain rates in individual. The construction of the chromosome is shown in Figure 4. 

**Figure 5:**
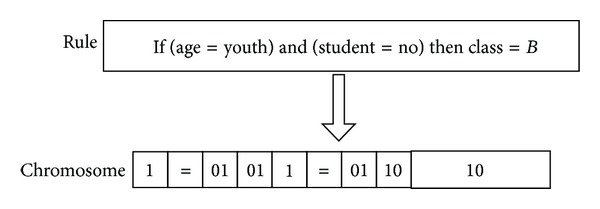
A simple example for rule coding.

In this method, although the length of the chromosome is fixed, the length of rule can be variable; it will mine out rules with more simplicity. As shown in Figure 5, it is a simple example of rule coding; if we have rule IF (*age* = *youth*) and (*student* = *no*) and then *Class* = *B*, then we can code it with “1 = 01011 = 011010.”

#### 4.2.2. Fitness Function for the Rule

In genetic algorithm, the fitness function is a measure to evaluate good or bad of the individual. In this paper we can divide the sample into four classes:

1. *T*_*T*: it denotes the number of the rule predicting the sample is true and the actual is true;
2. *T*_*F*: it denotes the number of the rule predicting the sample is true and the actual is fault;
3. *F*_*T*: it denotes the number of the rule predicting the sample is fault and the actual is true;
4. *F*_*F*: it denotes the number of the rule predicting the sample is fault and the actual is fault.

As shown before, we can set a variable to construct fitness function, we can call it accuracy. The formula of the accuracy is

(6) $$\begin{array}{c} \text{accuracy}=\frac{T\_T+F\_F}{T+F}, \end{array}$$

where *T* is the number of the sample which is true and *F* is the number of the sample which is fault. The accuracy can be the degree of accuracy the rule works on the training data. The higher the value is, the more samples correct classification.

Another variable is support; the formula of the support is

(7) $$\begin{array}{c} \text{support}=\frac{T\_T+F\_T}{T+F}. \end{array}$$

The lrager the value is, the greater proportion the rule in the data space; it means the rule has a better significance.

In this paper, we set the 3th variable to evaluate the fitness, named simplicity; the formula of the simplicity is

(8) $$\begin{array}{c} \text{simplicity}=\frac{N\left(\text{attributes}\right)-n\left(\text{rule}\;\text{attributes}\right)}{N\left(\text{attributes}\right)}, \end{array}$$

where *N*(*attributes*) is the number of attributes in the data set and *n*(*rules*_*attributes*) is the number of attributes in rule. The higher the simplicity of the individual, the simpler the rule, and the rule can be understood easier.

At last, the genetic algorithm will be used to produce the decision tree, so we should take the information gain ratio as a variable in the fitness function. The information gain ratio can be calculated with the formula as shown in Section 2 and we use gain ratio to express it. As analyzed above, the fitness function can be constructed as in following formula:

(9) Max⁡  fitness=a×simplicity+b×support +c×accuracy+d×Gain Ratio,

where *a*, *b*, *c*, and *d* are weight of the variables which in [0, 1] and *a* + *b* + *c* + *d* = 1.

#### 4.2.3. Crossover and Mutation Operations for the Rule

In this paper, we should select a sample *R* in training data where classification attribute is *C*_*i*_, randomly, and then code *R* to the individual coding string based on the code rules. In this way, the new generated individuals are effective individuals; it will reduce the search space of the algorithm greatly and improve the speed of the algorithm.

Two-point crossover is used to the chromosomal chiasma in this paper; firstly produce a random real number *Sc* which in [0, 1], if *Sc* is less than the crossover probability *Pc*, then select individuals  *a*_*i*_ and *a*_*j*_ randomly to crossover.

Produce a random real number  *Sm* which in [0, 1], if *Sm* is less than the mutation probability *P*_*m*_, we will do mutation on the individual. For the gene in this paper having four parts, so we must consider the gene construction sufficiently. So it will include three mutation operations (gain ratio gene will not change in these operations). 

**Figure 6:**
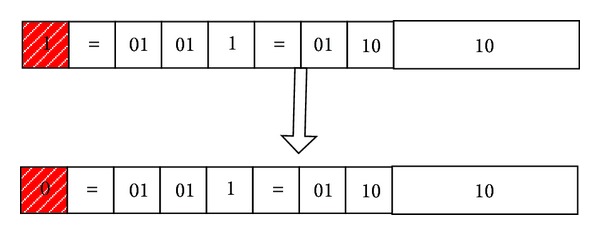
An example for weight mutation.

*Weight Mutation.* If the weight of original gene is 1, then mutate it to 0; if the weight of original gene is 0, then mutate it to 1. In this paper, we set that if the weight mutates from 1 to 0, the attribute which the gene corresponds to will not appear in the rule. For example, as shown in Figure 6, through the weight mutation, we can get the following rule:

(10) $$\begin{array}{c} \text{IF}\;\;\left(\text{student}=\text{no}\right)\;\;\text{then}\;\text{Class}=B. \end{array}$$

**Figure 7:**
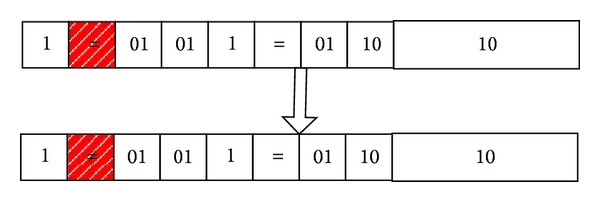
An example for operator mutation.

*Operator Mutation.* To the discrete attributes, if the operator of original gene is “=,” then mutate it to “≠”; if the operator of original gene is “≠,” then mutate it to “=.” To the continuous attributes, if the operator of original gene is “≥,” then mutate it to the “≺”; if the operator of original gene is “≺,” then mutate it to the “≥”; for example, as shown in Figure 7, through the operator mutation, we can get the following rule:

(11) $$\begin{array}{c} \text{IF}\;\left(\text{age}\neq \text{youth}\right)\;\;\text{and}\;\left(\text{student}=\text{no}\right),\text{then}\;\text{Class}=B. \end{array}$$

That is to say

(12) IF  (age=middle_age  or  age=senior)  and  (student=no),  then  Class=B.

**Figure 8:**
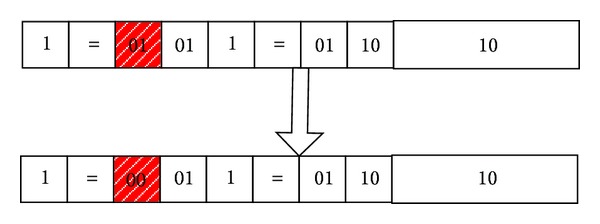
An example for value mutation.

*Value Mutation.* To the discrete attributes, choose a value in the attribute to replace the value in the original, randomly; to the continuous attributes, produce a decimal randomly and then do plus or minus on the original value with the decimal. For example, as shown in Figure 8, through value mutation, we can get the following rule:

(13) IF  (age=middle_age)and  (student=no),  then  Class=B.

Each mutation can be one mutation operation or any combination of the mutation operations.

### 4.3. Algorithm Description

The whole algorithm process flow can be described as follows.

**Step 1**

Initialize the population; a sample *R* with *S* records is randomly selected from the training set, whose class attribute value is *C*_*i*_. Then the evolution algebra variable num and the population initial average fitness variable *avg* are both assigned zero.

**Step 2**

Preprocessing operations were conducted on the sample *R*, including data cleaning, continuous attribute discretization, calculating the information gain rations of each feature attribute, and encoding the record data. Ultimately we have the initial encoded population *P*(*r*).

**Step 3**

Compute the fitness of each individual in the population, and then the average fitness is figured out.

**Step 4**

If the value of num is less than the maximum evolution population or *avg*_*i*_ − *avg*_*i*−1_ > *ε*, then repeat Steps 5, 6, and 7; otherwise go to Step 8.

**Step 5**

Calculating the average fitness of this generation, selection, crossover, and mutation operations are conducted on this population; thus offspring population is generated.

**Step 6**

Replace the individuals with low fitness in the parent by the ones that have high fitness in the offspring population; therefore, new generation is formed.

**Step 7**

Compute the fitness of each individual in the new generation, the average fitness as well.

**Step 8**

Those individuals whose fitness value is less than the lowest fitness threshold are taken out. The optimized population is the optimal set of rules.

**Figure 9:**
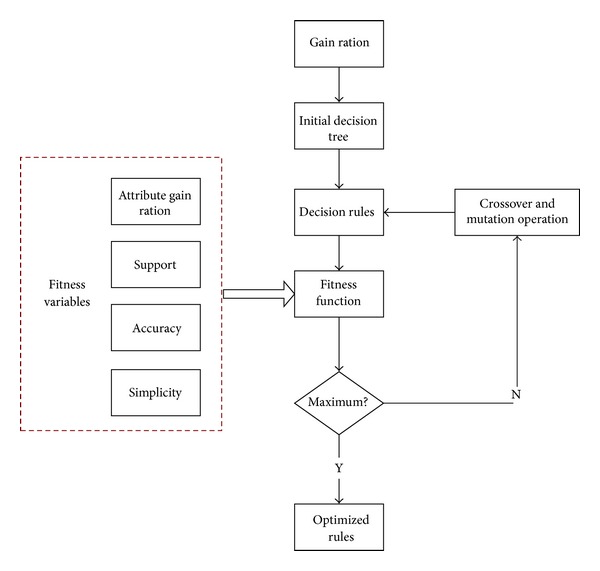
Modified decision tree algorithm processes.

The framework of the algorithm processes is shown in Figure 9 and the algorithm description is shown in Algorithm 2.

## 5. Mobile Customer Classification

We will use the modified decision tree algorithm based on genetic algorithm we proposed in this paper to deal with the mobile user classification firstly; then we will analysis the performance of the algorithm in this section.

### 5.1. Experiment

In order to verify this algorithm, we collected 1000 groups of the mobile user data and stored them in our data base. In this data set, ithas 6 feature attributes and one classification attribute; the information of these attributes is shown in Table 1.

We selected 70% of the data set as the training data and others as the test data. Then we set the parameter value as shown in Table 2.

Running the algorithm, we can get the rule as shown in Table 3.

As shown in Table 3, we can see that the most important factor which will influence the classification result is the attribute Wireless cost. If the Wireless cost is larger than 27.7 and under the public context, the user will belong to the Total service class with 84.66% accuracy in the training data and 83.25% in the test data. Then the less important factor attribute is the Free part; if Wireless cost is less than 27.7 and the Free part larger than 13.5 under the public context, the user will belong to the Plus service class with 72.68% accuracy in the training data and 70.13% in the test data; if  Wireless cost is less than 27.7 and the Free part is less than 13.5 and the user under private context and the moth is less than 16, the user will belong to Basic service; this rule has 73.07% accuracy in training data and 71.64% in the test data. If Wireless cost less than 27.7 and the Free is less than 13.5 and under the private context and month is larger than 16, the user will belong to E-service; the accuracy of this rule is 64.8% in training data and 63.79% in test data. So according to above analysis, the least important is month and the most important factor is Wireless cost.

### 5.2. Algorithm Analysis

In this section, we will analysis the algorithm we proposed in this paper. We will analysis the algorithm from two parts: one is comparison on the same data, the advantage of the algorithm and the other one is analysis of the performance of the algorithm.

To verity the effects of the method we proposed in this paper, we use C4.5 algorithm and SVM method to deal with the mobile user data set. We can get 5 rules with C4.5 algorithm and 6 rules with SVM algorithm. The average accuracy of each algorithm is shown in Table 4. We can see that the accuracy of C4.5 algorithm in the mobile user training data is 68.2% and 67.9% in the test data. The accuracy of SVM algorithm in the mobile user training data is 72.5% and 70.1% in the test data. We can conclude that the accuracy of C4.5 algorithm on the mobile user is lower than that of the SVM algorithm, but the rule which is generated by the SVM will be understood hardly.

Then we put the accuracy of three algorithms in one table, to show the advantage of our algorithm that we proposed. As we can see from Table 5, the accuracy of the DT-GA algorithm that we proposed in this paper is superior to C4.5 and SVM algorithms.

Another advantage of our algorithm is simplicity; in other words, the rule which is generated by our algorithm can be understood easily. Rules generated by other algorithms will be hardly. For example, the first rule in Table 5 is

(14) IF  (Wireless  cost≥27.7) and  (Context=public  context)   THEN  class=Total  service.

We can understand this rule very easily. The rule by SVM

(15) IF  (Wireless  cost≥27.7)  and  (Free  part≥0) and  (Context=public)  and  (month>0)THEN  class=Total  service.

This rule is too long to be understood. So we can conclude that the algorithm DT-GA that we proposed in this paper is super than other algorithm not only in accuracy but also in simplicity of the rule.

We do other experiments to show the algorithm we proposed in this paper; we used Iris and breast-cancer data sets, which are two main data sets of machine learning database UCI. The numbers of samples are 150 and 286, respectively; 4 characteristic attributes and 1 class attribute are included in the Iris database, 34 characteristic attributes and 1 class attribute are included in the breast-cancer database.

Two thirds of the data sets are randomly selected as training set and the rest as test set. The class is set as default class that has the most samples. And the discretization process in advance is undesired for continuous attributes in the experiment. At the same time, there is no additional process towards default value but filling the missing value with a negative value.

In this paper, the algorithm parameter values are shown in Table 6.

**Figure 10:**
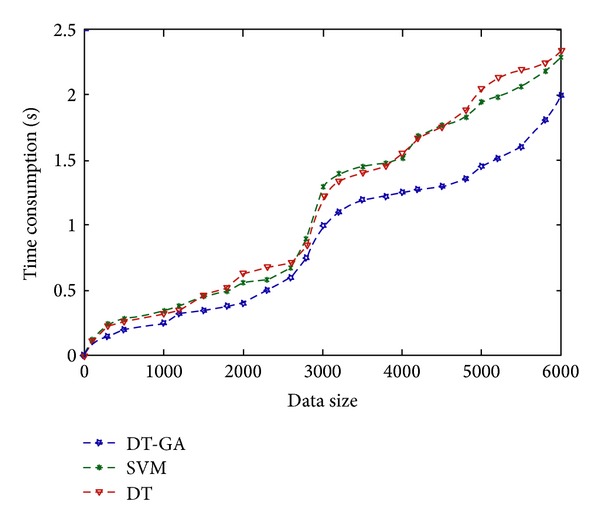
Time consumption comparison.

Through the experiment, by comparing the accuracy of algorithm C4.5, we can get the effectiveness of our method. Firstly, C4.5 algorithm is applied to the two data sets we mentioned before; the accuracy on the training set is 68.20% and 71.90%, respectively, and the accuracy of SVM method is 72.50% and 72.60%. Then we use the algorithm to deal with the test set; the accuracy on Iris test data is 70.10% and breast-cancer test set is 69.10%. However, the accuracy of our method on the Iris training set is 73.82% and the accuracy of the breast-cancer training set is 78.50%, respectively; the accuracy of our algorithm on the Iris test set is 72.20% and the accuracy on breast-cancer test set data is 76.4%. The accuracy is as shown in Table 7 and Figure 10 is the time consumption comparison with C4.5 and SVM algorithms.

The experiment, we can conclude that the algorithm we proposed in this paper has advantage on the accuracy and simplicity of the rule.

## 6. Conclusion

In this paper we proposed a modified decision tree based on genetic algorithm; it takes advantage of genetic algorithm optimization ability. We constructed the process of this algorithm firstly, and then we do an experiment with the algorithm; through the comparison, we can conclude that the algorithm which we proposed in this paper was improved compared to normal decision tree algorithm on accuracy. At last, we applied this algorithm on mobile users, and with the algorithm we can classify the mobile user into Basic service user, E-service user, Plus service user, and Total service user classes. Four rules with higher accuracy have been generated with the algorithm.

The further work will be on classifying the mobile user with more users' attributes and analyzing the performance of the algorithm, and we will use the algorithm to other fields if it possible, such as tourist classification, customer churn.

## Figures and Tables

**Algorithm 1:**
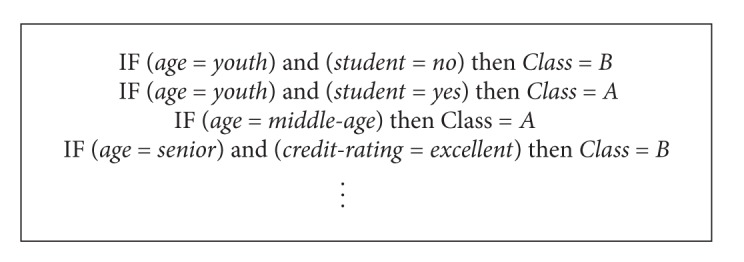
Expressions for Figure 2.

**Algorithm 2:**
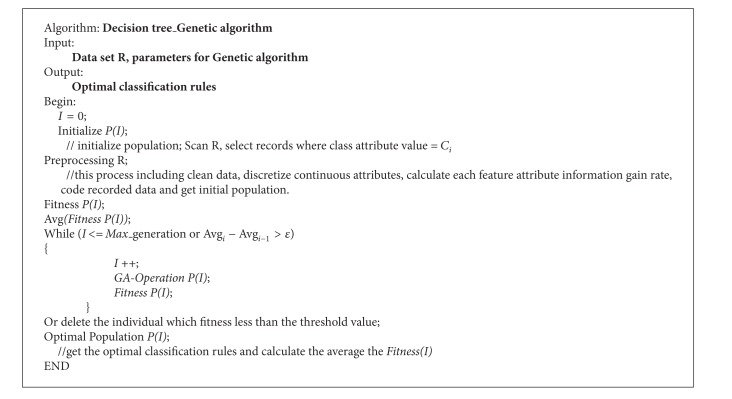
Algorithm description.

**Table 1:** Mobile user attributes information.

Attribute	Comment	Type	Value
Month	How many months did the user use the mobile service in?	Numeric	None
Wireless service	Did the user use the wireless service?	Boolean	0: no 1: yes
Basic cost	How much did the user spend on the mobile service?	Numeric	None
Free part	How much was the service to the user for free?	Numeric	None
Wireless cost	How much did the user spend on the wireless service?	Numeric	None
Context information	Which context is the user in?	Discrete	Private context Public context
Class	The class which the mobile user belong to.	Discrete	Basic service E-service Plus service Total service

**Table 2:** Parameter value for mobile user classification.

Parameter	Value
Crossover probability	0.85
Mutation probability	0.09
Maximum number of iteration	1000
Weighting coefficient	*a* = 0.1, *b* = 0.1, *c* = 0.1, and *d* = 0.7

**Table 3:** Rules on mobile users generated by the algorithm.

ID	Rule	Training set accuracy	Test set accuracy
1	IF (Wireless cost ≥ 27.7) and (Context = public context) THEN class = Total service	84.66%	83.25%
2	IF (Wireless cost ≤ 27.7) and (Free part > 13.5) and (Context = public context) THEN class = Plus service	72.68%	70.13%
3	IF (Wireless cost ≤ 27.7) and (Free part ≤ 13.5) and (Context = private) and (month ≤ 16) THEN class = Basic service	73.07%	71.64%
4	IF (Wireless cost ≤ 27.7) and (Free part ≤ 13.5) and (Context = private) and (month > 16) THEN class = E-service	64.87%	63.79%

**Table 4:** Compare C4.5 to decision tree based on genetic algorithm on accuracy.

Data set	Accuracy of C4.5 algorithm	Accuracy of SVM algorithm
Mobile user data		
Training data	68.2%	72.5%
Test data	67.9%	70.1%

**Table 5:** Comparison on accuracy.

	Training data	Test data
DT-GA	73.82%	72.20%
C4.5	68.20%	67.90%
SVM	72.50%	70.10%

**Table 6:** Experiment parameter value.

Parameter	Value
Crossover probability	0.8
Mutation probability	0.1
Maximum number of iteration	500
Weighting coefficient	*a* = 0.1, *b* = 0.1, *c* = 0.2, and *d* = 0.6

**Table 7:** Accuracy on Iris and Breast-cancer data.

	Iris test data	Breast-cancer test data
DT-GA	72.20%	76.40%
C4.5	67.90%	68.50%
SVM	70.10%	69.10%
